# Biotransformation and toxicokinetics of 2-phenoxyethanol after oral exposure in humans: a volunteer study

**DOI:** 10.1007/s00204-024-03717-2

**Published:** 2024-04-26

**Authors:** Elisabeth Eckert, Thomas Jäger, Julia Hiller, Edgar Leibold, Michael Bader, Thomas Göen

**Affiliations:** 1 Institute and Outpatient Clinic of Occupational, Social and Environmental Medicine, Friedrich-Alexander-Universität Erlangen-Nürnberg, Erlangen, Germany; 2 BASF SE, Corporate Health Management, Ludwigshafen, Germany; 3 Bavarian Health and Food Safety Authority, Erlangen, Germany; 4 BASF SE, Product Safety, Ludwigshafen, Germany

**Keywords:** 2-Phenoxyethanol, Urine, Blood, Biomonitoring, Metabolism, Toxicokinetics, Human exposure, In vivo study, Biocide, Oral, Dermal

## Abstract

2-Phenoxyethanol (PhE) is an aromatic glycol ether and is used in a variety of functions and applications, e.g., as preservative in pharmaceuticals, cosmetic and personal care products, as biocide in disinfectants (e.g. human hygiene), or as a solvent in formulations (e.g. coatings, functional fluids). Despite its widespread use, little is yet known on its biotransformation and toxicokinetics in humans. Therefore, a pilot study was conducted with oral administration of PhE (5 mg/kg body weight) to five volunteers. Blood and urine samples were collected and analyzed for PhE and three of its presumed metabolites up to 48 h post-exposure. Additionally, one volunteer was dermally exposed to PhE and monitored until 72 h post-exposure. PhE was rapidly resorbed following both oral and dermal application with *t*_max_-levels in blood of about 1 h and 3 h, respectively. Metabolism of PhE was observed to be rather extensive with phenoxyacetic acid (PhAA) and 4-hydroxyphenoxyacetic acid (4-OH-PhAA) as the main metabolites found in blood and urine following oral and dermal exposure. PhE was excreted rapidly and efficiently via urine mostly in metabolized form: following oral exposure, on average 77% and 12% of the applied dose was excreted within 48 h as PhAA and 4-OH-PhAA, respectively. A similar metabolism pattern was observed following the single dermal exposure experiment. The obtained data on biotransformation and toxicokinetics of PhE in humans provide valuable information on this important chemical and will be highly useful for pharmacokinetic modelling and evaluation of human PhE exposure.

## Introduction

2-Phenoxyethanol (PhE, CAS No. 122-99-6, synonym: ethylene glycol monophenyl ether), an amphiphilic organic compound often applied in mixtures of substances with different polarities, is an effective agent against Gram-negative and Gram-positive bacteria, yeast and mould. PhE is commonly used as a broad-spectrum preservative in cosmetics, pharmaceuticals or vaccine formulations (Dréno et al. 2019; SCSS 2016) as well as a biocidal component of metal-working fluids (Hartwig and MAK Commission 2019; ECHA 2023). According to the European Cosmetics Regulation No. 1223/2009, PhE is allowed to be used as a preservative in cosmetic products up to a maximum concentration of 1.0% in the European Union. Furthermore, it is used as a topical antiseptic in concentrations up to 2.0% in over-the-counter skin disinfectant formulations, e.g. Octenisept^®^ that is particularly used as disinfectant for the treatment of children’s wounds. Especially over the last years, increasing production volumes of PhE as well as an increasing number of PhE-containing products (non-cosmetics) were reported in a recent study (Liden et al. 2022).

Several animal studies concerning PhE toxicity following short-term and long-term exposure are available (Hartwig and MAK Commission 2019; SCSS 2016). The oral studies showed that rabbits are the most sensitive species to PhE, with the critical toxicological effect identified as haematotoxicity that was observed to be less pronounced in rats and mice (SCCS 2016). Follow-up in-vitro tests further showed that human red blood cells are more resistant to lysis than those of other species. Accordingly, target organs of PhE toxicity in rats and mice following oral exposure were liver and kidney instead (showing histopathological changes). PhE showed no carcinogenic activity in rats and mice and was found to be not genotoxic in several studies (reviewed by Hartwig and MAK Commission 2019; SCSS 2016). As dermal exposure is expected to be the main exposure route of PhE in humans, a dermal study in rabbits (as the most sensitive species) was selected by the SCCS (2016) as a key study, and a NOAEL (no observed adverse effect level) of 357 mg/kg body weight (bw) and day was identified. However, due to the known higher capacity of humans to metabolize PhE, a reduced assessment factor of 25 was selected to extrapolate this NOAEL to human exposure leading to a derived health based guidance value for dermal exposure to PhE of 14.28 mg/kg bw and day (SCCS 2016). During the ECHA registration process of PhE similar DNEL (derived no effect levels) of 10.42 mg/kg bw and day and 9.23 mg/kg bw and day for dermal and oral exposure to PhE of the general population, respectively, were identified (ECHA 2023).

Despite the long-term use of PhE in various consumer goods, surprisingly little is known about its biotransformation pathways and its toxicokinetic behavior in humans. Based on animal studies conducted with ^14^C-labelled PhE, PhE is known to be rapidly and almost completely absorbed (bioavailability of 75% to 98%) following oral exposure. Absorbed PhE is then extensively metabolized and mainly excreted in urine with phenoxyacetic acid (PhAA) or its conjugates as the main metabolites observed in humans and animals (ECHA 2023; Hartwig and MAK Commission 2019; SCSS 2016). The metabolism of PhE was further investigated in female rats after oral administration (ECHA 2023; Hartwig and MAK Commission 2019; Kim et al. 2015): Here, PhAA was identified as the main metabolite in urine, that was excreted primarily unconjugated in amounts of 57–74% within 24 h, whereas glucuronidated PhAA in urine only accounted for about 5% of the administered dose. Furthermore, ring-hydroxylated metabolites were found and urinary excretion percentages of 8–10% were calculated for these metabolites. However, no more details regarding the exact identification of those ring-hydroxylated metabolites were given. Unmetabolized PhE was described to be excreted in urine in very low levels of less than 1%. All in all, urinary elimination following oral administration accounted for over 90% of the dose, irrespective of the exposed amount, and PhAA was the major metabolite. For rats, initial half-lives of PhE and its metabolites in plasma of 1.9–4.6 h were reported.

Few biomonitoring studies on human exposure to PhE are yet available. These focused on the determination of PhAA as the presumed main human metabolite of PhE (Fromme et al. 2013; Garlantezec et al. 2012; Labat et al. 2008; Multigner et al. 2007). However, due to the lack of detailed information regarding human metabolism of PhE and its toxicokinetics, the following issues still remain unclear: (1) the urinary excretion factor of PhAA in humans, (2) the urinary elimination rate of PhAA in humans, and (3) the question whether further urinary human metabolites of PhE have to be considered.

**Fig. 1:**
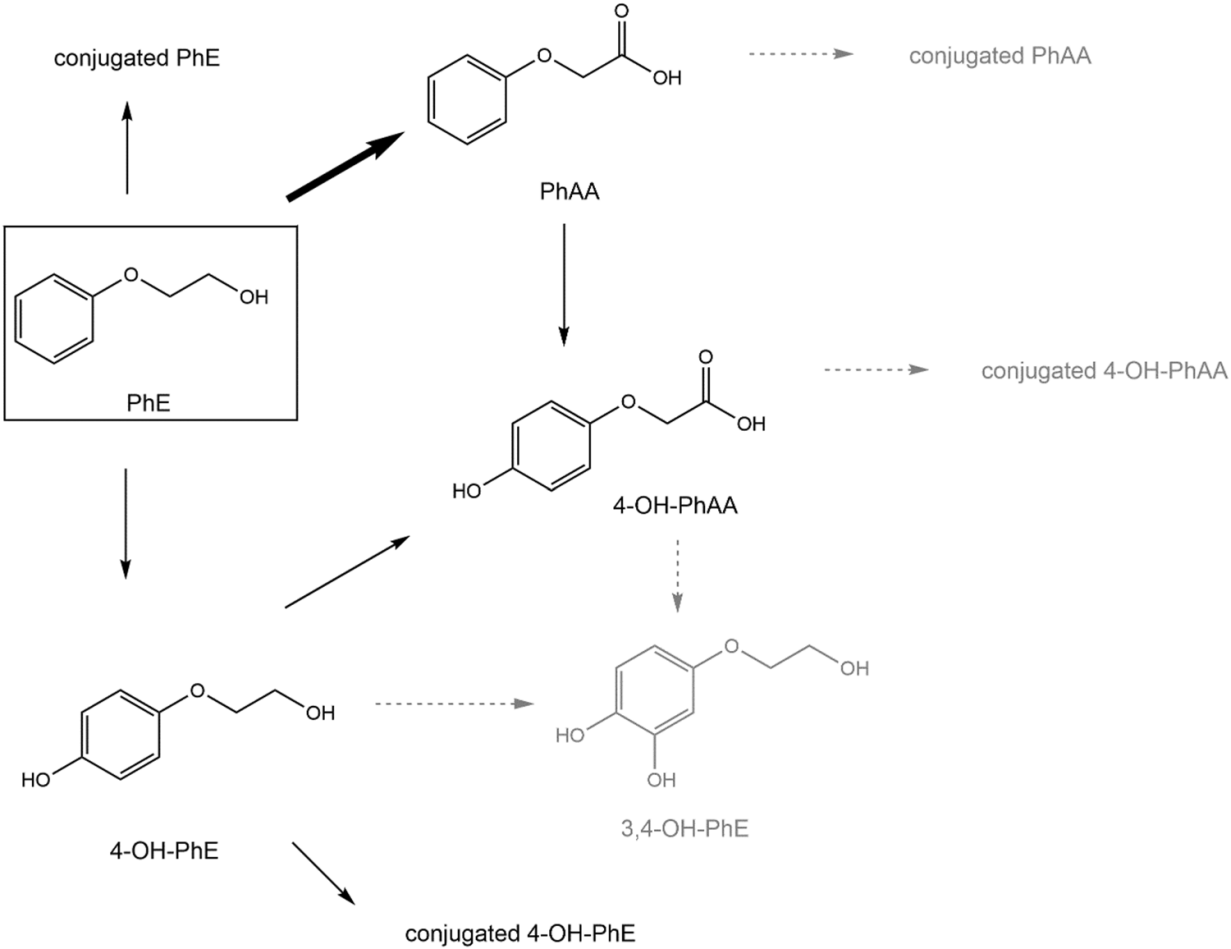
Proposed metabolism scheme of PhE in humans (3,4-OH-PhE: 3,4-dihydroxy-phenoxyethanol)

Due to its widespread use and the potentially high consumer exposure, PhE was recently selected as a chemical of interest in a collaboration project for the improvement of human biomonitoring between the German Federal Ministry for the Environment, Nature Conservation, Nuclear Safety and Consumer Protection (BMUV) and the German Chemical Industry Association (VCI). The basic goal of this project is to enable exposure assessment of the general population for substances of potential concern, e.g. due to their toxicity or their relevance for consumer exposure, by establishing reliable human biomonitoring methods and to investigate the biotransformation pathways including the main toxicokinetic parameters of the substances of interest. Thus, recently, biomonitoring methods for the determination of PhE and its metabolites in human blood and urine were developed (Jäger et al. 2022, 2024). In this context, background levels of PhE and further potential PhE metabolites, namely 4-OH-PhE (4-hydroxy-phenoxyethanol) and 4-OH-PhAA (4-hydroxy-phenoxyacetic acid), were assessed for the first time in human blood and urine, respectively. Figure 1 shows the proposed biotransformation pathways of PhE in humans based on these preliminary results.

Recently, several studies focused on PBPK modelling to predict tissue and urine concentration of PhE following oral, dermal or inhalation exposure (Kwon et al. 2021; Troutman et al. 2015; Zhang et al. 2022). The results, however, vary considerably and leave many issues unresolved. These models are therefore of limited utility to predict biotransformation and toxicokinetics of PhE in humans.

Thus, the aim of the present study was the investigation of PhE metabolism in humans following single oral exposure of five volunteers. Furthermore, the results were compared to a single experiment, where one volunteer was additionally dermally exposed to PhE using the same administration dose.

## Materials and methods

### Study population and study design

The study was approved by the local ethics committee of the Friedrich-Alexander-University of Erlangen-Nürnberg, Germany (No. 296_19 B). All participants were informed about the aims and risks of the study and gave their written informed consent to their participation. All subjects were healthy adults who were not occupationally exposed to PhE. The volunteers were instructed to avoid potentially PhE-containing consumer products for at least three days prior to PhE administration and during the whole time of sample collection following PhE administration.

The study collective consisted of five volunteers (2 males, 3 females) with a median age of 30 years (range 28–56 years) and a median body weight of 84 kg (53–117 kg). All volunteers were orally exposed with a single dose of 5.02 ± 0.10 mg PhE/kg bw, corresponding to an absolute dose of 423.8 ± 111.2 mg PhE. For oral exposure, PhE was weighed exactly on a small rusk covered with chocolate on the bottom side. It was taken care that the rusk was consumed all at once by each volunteer. One volunteer (male, 56 years, 87 kg) was additionally exposed dermally with PhE in a single experiment with an absolute dose of 430.4 mg (4.95 mg/kg bw). This was done by non-occlusive application of an ointment (basic formulation) containing 10% PhE on the volunteer’s back (area of 2000 cm^2^). The application site was left uncovered for more than 6 h after administration to enable complete absorption of the ointment and to avoid any artificial losses.

The oral and dermal administration of PhE took place during November 2019 and February 2021. The oral and dermal dose was selected to fall safely below the respective DNEL values of the ECHA which are set at 9.23 mg/kg bw and day and 10.42 mg/kg bw and day for acute/short-term oral exposure to PhE and long-term dermal exposure to PhE, respectively (ECHA 2023).

Prior to exposure, each volunteer was asked to deliver one spot urine sample. Additionally, one blood sample was drawn using EDTA monovettes (pre-exposure samples). The PhE administration was performed early in the morning and the volunteers were instructed to collect all urine samples up to 48 h post-exposure. During the first 8 h after exposure, the volunteers were asked to provide urine samples, if possible, in an hourly interval. All urine samples were weighed to enable an accurate estimation of the individual urine volumes and were stored frozen at − 20 °C until analysis. Additionally, blood samples were drawn in regular intervals from each volunteer at 1, 2, 3, 4, 6, 10, 24, and 48 h post-exposure and were also stored frozen at − 20 °C until analysis. One volunteer was asked to collect blood and urine samples up to 72 h post-exposure (cf. Table 1).

**Table 1:** Subject characteristics and number of collected samples

Subject No	Age [years], sex	Body weight [kg]	PhE dose [mg (mg/kg bw)	Sampling time [h]	No. of urine samples	Total urine volume [L]	No. of blood samples
Oral							
1	56, m	87	423 (4.86)	72	42	5.65	13
2	29, f	84	425 (5.06)	48	28	2.10	10
4	28, m	117	582 (4.98)	48	22	2.46	10
5	30, f	53	268 (5.05)	48	22	7.41	10
6	33, f	82	421 (5.13)	48	27	2.34	10
Dermal							
1	56, m	87	430 (4.95)	72	42	3.96	14

### Analytical determination of PhE and its metabolites in blood and urine

The analytical determination was done according to previous published procedures by Jäger et al. (2022, 2024). Thus, all reagents and chemicals used as well as the applied sample preparation procedures and the instrumentation used is described in detail there.

Briefly, the PhE metabolites PhAA and 4-OH-PhAA were determined using LC–MS/MS analysis following sample preparation using a “dilute-and-shoot”-technique for urine analysis and liquid–liquid extraction for the blood samples. Preliminary experiments revealed that both, PhAA and 4-OH-PhAA, are excreted mainly unconjugated in human urine and that the conjugated forms are present in negligible amounts only. Thus, all analyses were done without an additional hydrolysis step (cf. Jäger et al. 2022). Unmetabolized PhE and 4-OH-PhE were determined using GC-PCI-MS/MS-analysis following liquid–liquid extraction and silylation. Here, a hydrolysis step was included as it was shown that both, PhE and 4-OH-PhE are excreted in significant amounts as conjugates (cf. Jäger et al. 2024). LOQ levels of PhAA, 4-OH-PhAA, PhE and 4-OH-PhE were 10, 20, 1.0 and 0.5 µg/L (urine) and 6, 10, 2.0 and 2.0 µg/L (blood), respectively.

### Data evaluation and statistical analyses

Data evaluation and statistical analyses were done using Microsoft^®^ Excel^®^ 2016 and Origin^®^ 2019, respectively.

Renal excretion rates (*R*_E_, in μg/h) of each analyte were calculated using the following equation:

$${R}_{E,i}=\frac{{c}_{i}\times {V}_{i}}{{t}_{i}-{t}_{i-1}},$$

where *c*_*i*_ is the analyte concentration in the urine sample (in µg/L), *V*_*i*_ is the volume of the urine sample (in L), *t*_*i*_ is the sampling time of the urine sample after exposure (in h) and *t*_*i−1*_ is the sampling time of the previous urine sample (in h).

Excretion curves were prepared for each study participant and each analyte by plotting the calculated excretion rates against the average time of the sampling period. Mean excretion curves were obtained using default sampling times for each participant and the corresponding mean renal excretion rates at this time point.

The ln-transformed mean excretion curve of each analyte was plotted against the time post-exposure (in h) to obtain the slope (*k*_el_, elimination rate constant) and the excretion half-life (*t*_1/2_) in blood and urine, respectively, as follows:

$${t}_{1/2}=\frac{{\text{ln}}(2)}{\left|{k}_{{\text{el}}}\right|}.$$

Urinary excretion factors (*F*_UE_) were expressed as PhE dose equivalents (in %) to evaluate the total excretion rate of recovered PhE and its metabolites in urine after 24 h and 48 h, respectively, based on the administered exposure dose of PhE using the following equation:

$${F}_{{\text{UE}}}=\frac{{{\text{CE}}}_{i}}{{{\text{M}}}_{D}}\times 100,$$

where CE_*i*_ is the mean cumulative amount of the respective analyte (in µmol) and *M*_D_ is the mean administered PhE dose (in µmol), respectively.

## Results

### Kinetics in blood after oral PhE administration

**Fig. 2:**
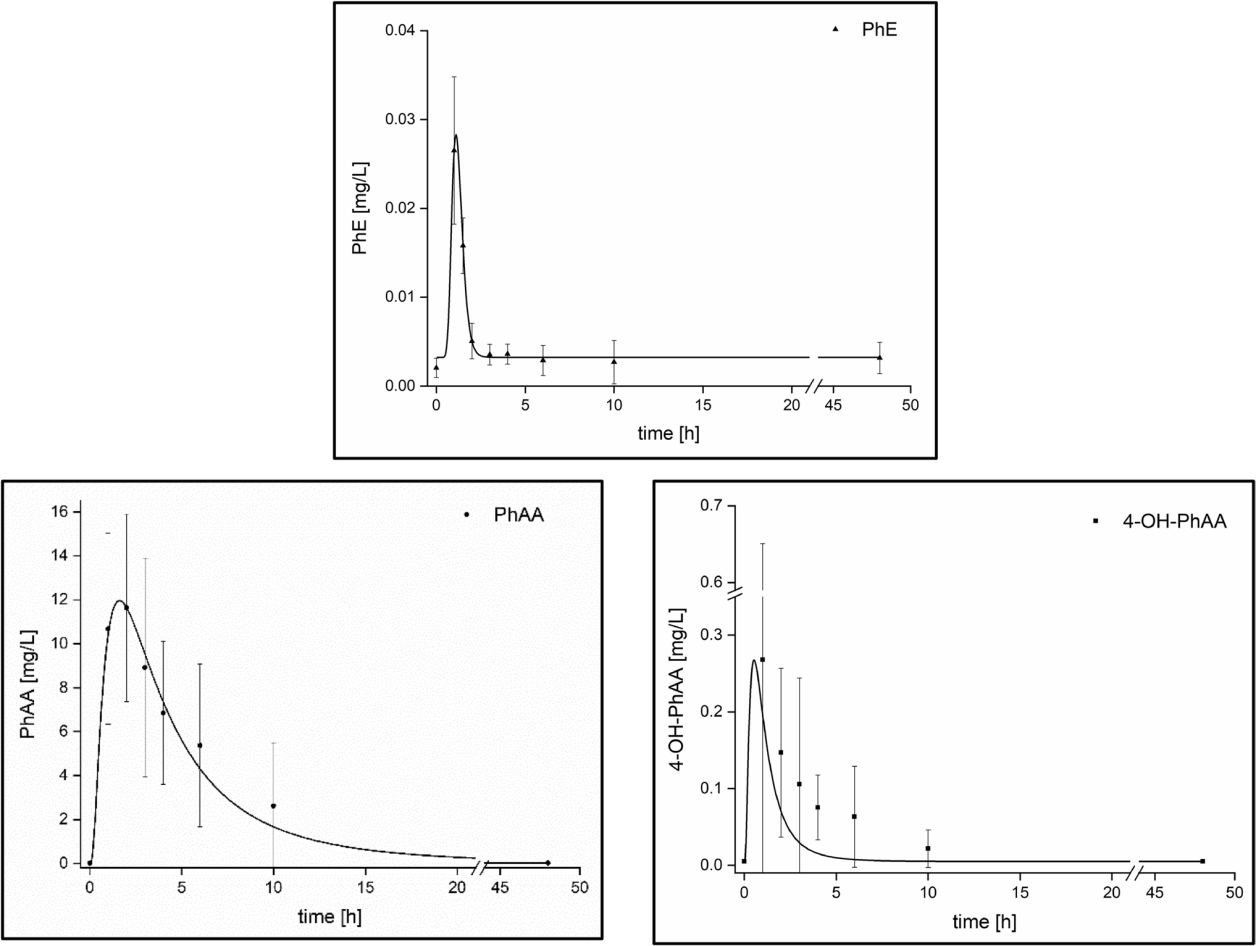
Excretion kinetics of PhE and two of its metabolites in blood following oral administration of about 5 mg/kg body weight PhE to five volunteers

The results for the kinetics of PhE and its metabolites in blood after oral administration are given in detail in Table 2. Following oral administration, elevated levels of unmetabolized PhE were found in blood during a period of up to 4–6 h post-exposure with a *t*_max_ level of 1 h. Apart from the parent compound, the presumed metabolites 4-OH-PhAA and PhAA were usually detectable in the blood samples up to 24 and 48 h post-exposure, respectively. The metabolite 4-OH-PhE was not found in any of the analyzed blood samples in levels above the LOQ. In comparison to PhE, slightly delayed *t*_max_ values were observed for the metabolites PhAA and 4-OH-PhAA, respectively. Quantitatively, the detected PhE levels were observed to be rather low with a *c*_max_ level of 0.027 ± 0.008 mg/L. Distinctly higher *c*_max_ levels combined with prolonged detection periods post-exposure were observed for the metabolites PhAA and 4-OH-PhAA. Figure 2 shows the average excretion kinetics of PhE and its metabolites PhAA and 4-OH-PhAA in blood.

**Table 2:** PhE excretion kinetics in blood following oral PhE administration to five volunteers

Analyte	*c*_max_ [mg/L]	*t*_max_ [h]	*t*_1/2_ [h]
PhE	0.027 ± 0.008	1.0 ± 0.2	1.7 ± 1.0
PhAA	12.9 ± 4.1	1.8 ± 0.4	3.5 ± 0.6
4-OH-PhAA	0.30 ± 0.37	1.3 ± 0.3	3.9 ± 1.4

### Urinary excretion kinetics after oral PhE administration

PhE was excreted in urine as PhE itself as well as in form of metabolites. Detailed characteristics of the urinary excretion data of PhE are summarized in Table 3. The administered PhE dose was excreted almost completely in urine within 48 h post-exposure, with PhAA as the main urinary metabolite accounting for about 75% of the applied oral dose. The ring-oxidized metabolite 4-OH-PhAA presented itself as another significant PhE metabolite and was excreted in urine corresponding to 12% of the oral PhE dose within 48 h on average. Interestingly, 4-OH-PhAA showed significant interindividual differences with regard to the urinary excretion rate. In two volunteers (both female) 4-OH-PhAA accounted for 19.5% and 15.8% of the oral PhE dose within 48 h post-exposure, respectively. This was approximately twice as much as for the other subjects (one female, two males), where *F*_UE_-levels of about 8% each, were observed.

**Table 3:** Renal excretion kinetics of PhE following oral administration of about 5 mg/kg bw to five volunteers

Analyte	*R*_E,max_ [mg/h]	*t*_max_ [h]	*t*_1/2_ [h]	*F*_UE_ [24 h, %]	*F*_UE_ [48 h, %]
PhE	0.50 ± 0.44	1.0 ± 1.3	1.3 ± 0.9	0.23 ± 0.10	0.23 ± 0.11
4-OH-PhE	0.16 ± 0.16	1.0 ± 1.3	1.4 ± 0.3	0.08 ± 0.06	0.08 ± 0.06
PhAA	54.2 ± 18.8	2.2 ± 0.9	4.9 ± 0.8	71.4 ± 20.0	76.7 ± 14.7
4-OH-PhAA	15.6 ± 13.6	2.0 ± 0.7	4.6 ± 0.9	11.4 ± 4.9	12.1 ± 5.3
Sum				83.1 ± 17.5	89.0 ± 11.8

Unmetabolized PhE as well as the hydroxylated metabolite 4-OH-PhE, were found in urine only to a minor extent with levels of < 0.5% each of the oral dose within 48 h post-exposure.

**Fig. 3:**
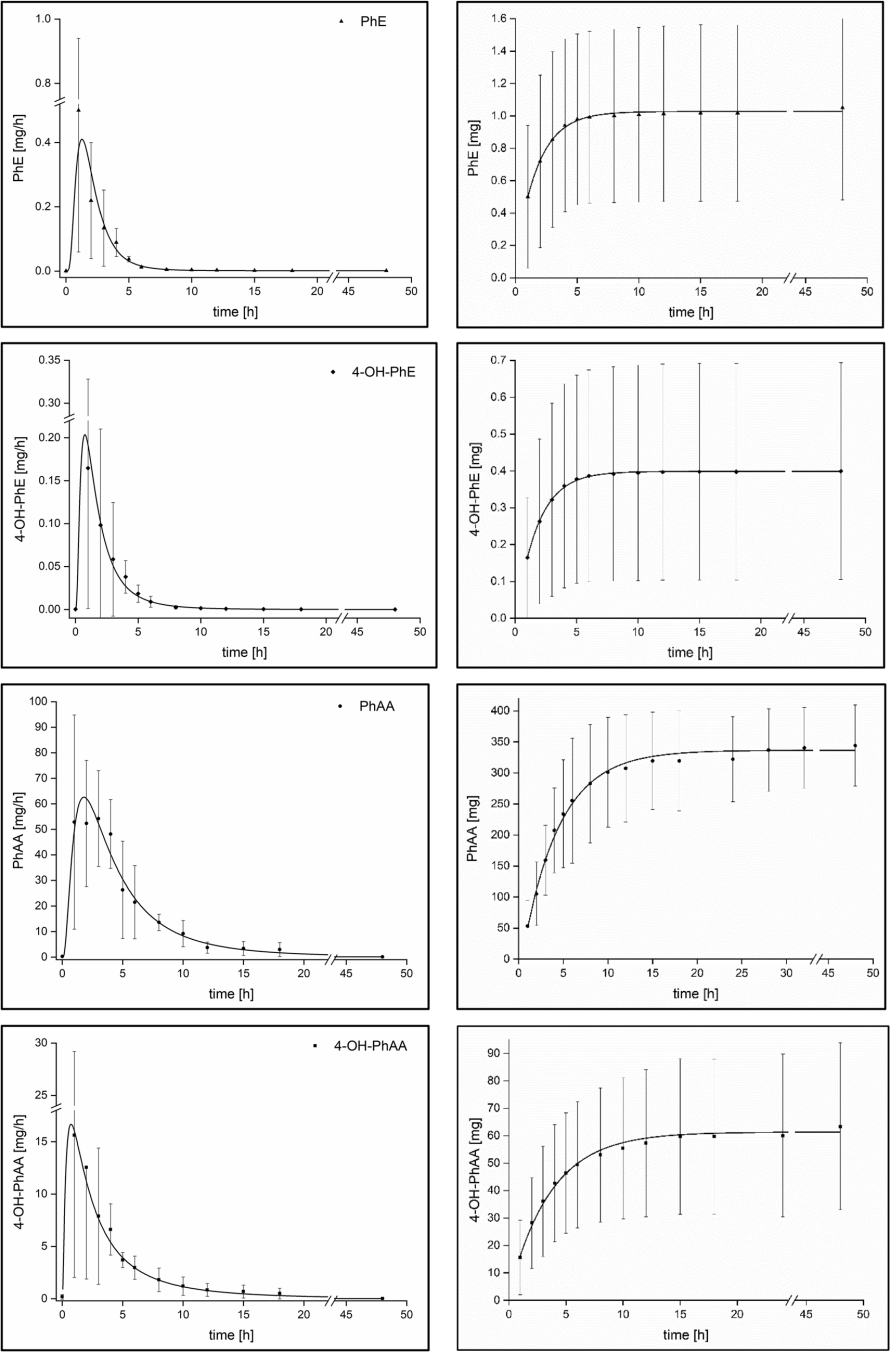
Urinary excretion kinetics of PhE and its metabolites following oral administration of about 5 mg/kg body weight PhE to five volunteers

The sum of *F*_UE_ for all analytes within 24 and 48 h post-exposure amounted to 83.1 ± 17.1% and 89.0 ± 11.8%, respectively. A prolonged observation time of 72 h post-exposure for one volunteer revealed an insignificant further increase of the *F*_UE_-level for all analytes of only 0.3% in comparison to the *F*_UE_-level 48 h post-exposure. In relation to all analytes determined in urine, PhAA and 4-OH-PhAA accounted for 85.7 ± 6.9% and 13.9 ± 6.7%, respectively (sum of all analytes set as 100% at 48 h post-exposure). PhE and 4-OH-PhE were observed to have distinctly shorter urinary excretion half-lives than PhAA and 4-OH-PhAA. Figure 3 shows the mean urinary excretion rates of PhE and its metabolites (left-hand side) as well as the cumulative excretion rates of all analytes (right-hand side).

### Single dermal PhE administration

**Fig. 4:**
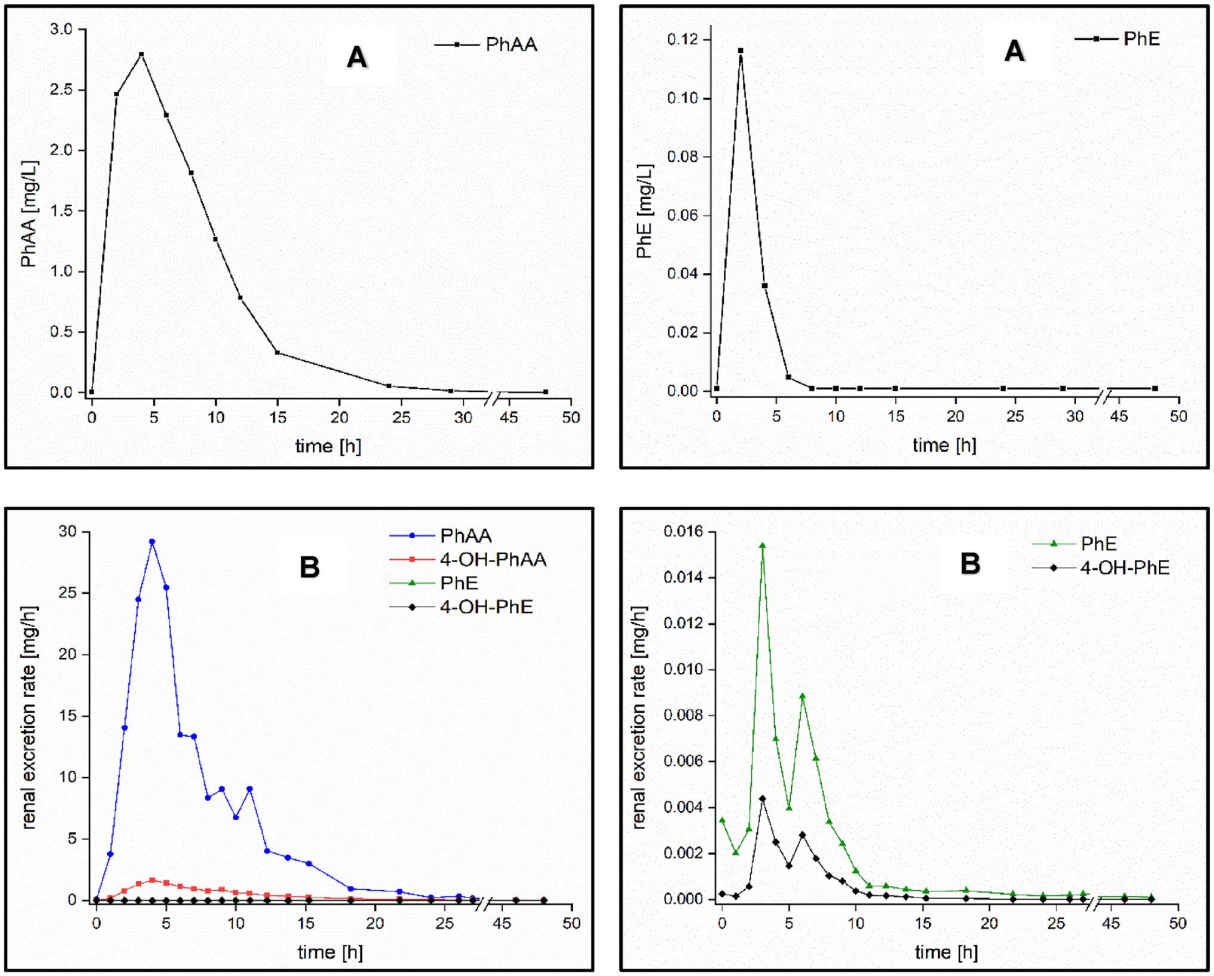
Excretion kinetics of PhE and its metabolites following single dermal administration of about 5 mg/kg body weight PhE: A) Excretion kinetics in blood; B) urinary excretion kinetics of PhE and its metabolites

Dermal exposure of one volunteer also showed a rapid resorption of PhE with *t*_max_ levels in blood and urine after 3–4 h post-exposure, respectively. In blood, elevated PhAA levels were mainly found up to 24 h post-exposure (cf. Figure 4), while 4-OH-PhAA was detectable in one sample only (4 h post-exposure). 4-OH-PhE was not found in any of the blood samples in levels above the LOQ. Unmetabolized PhE, however, was observed in levels above the LOQ up to 6 h post-exposure with *t*_max_ at 2 h and a *c*_max_ level of 116.3 µg/L. In comparison to the observed PhE blood levels following oral exposure of this volunteer it is striking, that the observed *c*_max_ level of unmetabolized PhE is higher about a factor of 4 following dermal exposure, while at the same time, significantly lower *c*_max_ levels of the metabolites PhAA and 4-OH-PhAA were observed following dermal exposure.

In urine, all analytes were detectable with PhAA as the main metabolite (cf. Fig. 4). Urinary elimination half-lives of 1.8 h, 1.9 h, 3.1 h and 4.2 h were observed for PhE, 4-OH-PhE, PhAA and 4-OH-PhAA, respectively. The sum of all analytes renally excreted within 48 h post-exposure amounted to 40.2% of the dermally applied amount. Again, a prolonged observation time up to 72 h post-exposure did increase the recovery rate only marginally (about 0.04%). For PhAA and 4-OH-PhAA an *F*_UE_ level of 37.8% and 2.4% was observed, respectively, while PhE and 4-OH-PhE were excreted in amounts of only < 0.05% each of the dermally applied dose. In relation to all determined analytes, PhAA and 4-OH-PhAA thus accounted for 93.9% and 6.0% of the dermally applied amount, respectively. Following oral PhE exposure of this volunteer, PhAA and 4-OH-PhAA showed very similar *F*_UE_ levels of 91.6% and 8.3%, respectively.

## Discussion

In the present study, the fast uptake and high bioavailability of PhE (as reported by many animal studies) could be confirmed for humans as was shown by the rapid occurrence of PhE and two of its metabolites in blood with mean *t*_max_ levels of 1.0–1.8 h depending on the analyte. The analyzed PhE metabolites showed (as presumed) a temporal delay in comparison to unmetabolized PhE with mean *t*_max_ levels of 1.3 h and 1.8 h for 4-OH-PhAA and PhAA, respectively. Levels of unmetabolized PhE in blood, however, were observed to be rather low with a mean *c*_max_ level of 0.027 mg/L and were found to be elevated during the first 6 h following oral administration only.

Up to now, only one other study investigated human metabolism of PhE following oral exposure by means of a volunteer study. Howes (1988) (reviewed by SCCS 2016) administered 10.3 mg of PhE to a single male adult volunteer and collected urine samples up to 72 h post-exposure. Based on an observed urinary recovery rate of 104%, complete absorption of PhE was postulated, whereas in urine only PhAA (and its conjugates) was detected. The present study confirms the high absorption rate of PhE after oral intake indicated by a mean relative urinary recovery rate of 89.0 ± 11.8% within 48 h post-exposure. PhE was also shown to be rapidly eliminated following systemic uptake showing an extensive urinary excretion rate. The main portion of absorbed PhE was observed to be eliminated via urine during the first 6–8 h following oral administration. Thus, the cumulative excretion curves presented in Fig. 3 illustrate that a plateau is reached within 10–15 h for all analytes implying a virtually completed elimination process for these analytes, thus confirming that a significant further PhE intake during the study did not become apparent.

Metabolism of PhE was confirmed to be fast and rather extensive with PhAA as the main metabolite found in blood and urine. PhAA is presumably formed through the conversion of the alcohol function of PhE to an aldehyde by alcohol dehydrogenase (ADH) followed by the conversion to an acid function catalyzed by aldehyde dehydrogenase (ALDH) (Hewitt et al. 2022; Hartwig and MAK Commission 2019; Lockley et al. 2005). However, in contrast to previous animal studies and the above mentioned human study, 4-OH-PhAA was identified as a further significant PhE metabolite excreted in urine with an absolute dose percentage of 8.0–19.5% within 48 h following oral exposure. 4-OH-PhAA may be formed by successive oxidation of PhAA or following side-chain oxidation of 4-OH-PhE (cf. Fig. 1). The identification of 4-OH-PhAA as a significant human metabolite of PhE is an important finding of the present study and represents a significant distinction to a recent PBPK model introduced by Hewitt et al. (2022), who concluded that apart from PhAA no further PhE metabolites had to be taken into account as they do not represent more than 10% of the total PhE metabolites and are therefore to be considered as minor metabolites only. It is recommended that substances representing more than 10% of the total metabolites are to be investigated further for potential toxicity (FDA 2020). In the present study, 4-OH-PhAA was found with a mean relative percentage of 13.9 ± 6.7% and a maximum value of even 23.2% in one volunteer after oral exposure. The presence of ring-hydroxylated metabolites of PhE, like 4-OH-PhAA, had been postulated by several other studies (reviewed by SCSS 2016), however up to now an exact identification or quantification was not performed. Additionally, in the present study, we were also able to quantify unmetabolized PhE and hydroxylated PhE (4-OH-PhE) in some of the urine samples of the volunteers following oral exposure. However, their *F*_UE_ levels were found to be rather low with mean dose percentages of 0.23 and 0.08% (up to 48 h post-exposure), respectively. This confirms the observation of other animal studies, where unmetabolized PhE was found in urine not at all or only in very low levels of < 0.7% (SCCS 2016).

Due to the major use of PhE in consumer products, the main exposure route for the general population is expected to occur via dermal contact. Therefore, an additional experiment was performed applying dermal PhE administration to one volunteer by use of a similar PhE body dose (about 5 mg/kg bw) as in the oral experiment. All in all, PhE resorption, metabolism and excretion following dermal exposure were observed to be rather similar in comparison to the results following oral exposure. Though, (as expected) a prolonged resorption time was observed with *t*_max_ levels in urine and blood of 3.0–4.0 h, respectively, compared to 1.0 to 2.2 h after oral exposure. Thus, the dermal absorption kinetics of PhE are well in line with the behavior of other amphiphilic compounds, e.g. *N*-methyl-2-pyrrolidone (Bader et al. 2005), whereas other more lipophilic chemicals (e.g. UV absorbers and polycyclic aromatic hydrocarbons) usually show a substantially longer resorption delay following dermal exposure with *t*_max_ levels in the range of 10–15 h (Hiller et al. 2019a, Hiller et al. 2019b; Viau and Vyskocil 1995). Furthermore, the urinary recovery rate following dermal exposure was observed to be significantly lower by a factor of about 2 compared to oral exposure probably due to a reduced dermal resorption rate of PhE. The bioavailability rate of PhE after single dermal exposure of about 40% is well in the range of dermal resorption rates reported or predicted by other studies (SCCS 2016; Kwon et al. 2021). However, in an animal study using occlusive dermal PhE application, a higher resorption rate of about 75% was observed (Kim et al. 2015). Striking is the difference regarding the observed PhE levels in blood. Following dermal exposure PhE was detectable in significantly higher levels (by a factor of 4) than after oral exposure. This is in line with animal studies where higher systemic PhE levels were consistently observed following intravenous or dermal exposure (SCCS 2016) and might be due to a significant first-pass effect following oral exposure preventing unmetabolized PhE to reach systemic circulation. However, PhE levels in blood were always observed to be substantially lower than the levels of PhAA. The toxicological significance of this effect, however, has to be evaluated in further studies. To this end, it is deemed to be necessary to collect more data of PhE toxicokinetics in humans, preferably following dermal exposure and using PhE doses that reflect PhE exposure levels of the general population.

## Conclusion

The present study demonstrates that PhE is rapidly and almost completely resorbed by the human body following oral exposure. It was shown to be quickly and extensively metabolized to PhAA, the main metabolite found in blood and urine. For the first time, 4-OH-PhAA and 4-OH-PhE were identified as further human metabolites of PhE, whereas 4-OH-PhAA was found in significant amounts of above 10% on average. The individual excretion rate of 4-OH-PhAA, however, was observed to vary considerably potentially due to interindividual differences in PhE metabolism as the volunteer who was exposed both orally and dermally, showed a very similar relative elimination rate of urinary 4-OH-PhAA, irrespective of the administration route. However, this hypothesis has to be pursued in further studies. Urinary elimination was found to be extensive and quite fast. Interestingly, PhAA as the main metabolite showed the longest urinary elimination half-life of 4.9 h in comparison to the other here considered analytes which supports its good suitability as biomarker of exposure to PhE. A single dermal exposure experiment confirmed the fast uptake of PhE into the human body, whereby a very similar metabolism pattern of PhE was observed with PhAA and 4-OH-PhAA as the main metabolites in urine, though showing a significant lower urinary recovery rate probably due to a reduced dermal resorption rate compared to oral resorption. The obtained data on biotransformation and toxicokinetics of PhE in humans provide valuable information on this important chemical and will be highly useful for pharmacokinetic modelling and evaluation of human PhE exposure.

## Data Availability

The data that support the findings of this study are available from the corresponding author, Elisabeth Eckert, upon reasonable request.

## References

[CR1] Bader M, Keener SA, Wrbitzky R (2005). Dermal absorption and urinary elimination of *N*-methyl-2-pyrrolidone. Int Arch Occup Environ Health.

[CR2] Dréno B, Zuberbier T, Gelmetti C, Gontijo G, Marinovich M (2019). Safety review of phenoxyethanol when used as a preservative in cosmetics. J Eur Acad Dermatol Venereol.

[CR3] ECHA (European Chemicals Agency) (2023) 2-Phenoxyethanol. Last updated 11/07/23. https://echa.europa.eu/de/brief-profile/-/briefprofile/100.004.173. Accessed 17 Jul 2023

[CR4] FDA (Food Drug Administration) (2020) Safety testing of drug metabolites guidance for industry. https://www.fda.gov/media/72279/download

[CR5] Fromme H, Nitschke L, Boehmer S, Kiranoglu M, Göen T (2013). Exposure of German residents to ethylene and propylene glycol ethers in general and after cleaning scenarios. Chemosphere.

[CR6] Garlantézec R, Multigner L, Labat L, Bonvallot N, Pulkkinen J, Dananché B, Monfort C, Rouget F, Cordier S (2012). Urinary biomarkers of exposure to glycol ethers and chlorinated solvents during pregnancy: determinants of exposure and comparison with indirect methods of exposure assessment. Occup Environ Med.

[CR7] Hartwig A, MAK Commission (2019). 2-Phenoxyethanol. MAK value documentation. Collect Occup Health Saf.

[CR8] Hewitt NJ, Troutman J, Przibilla J, Schepky A, Ouédraogo G, Mahony C, Kenna G, Varçin M, Dent MP (2022). Use of in vitro metabolism and biokinetics assays to refine predicted in vivo and in vitro internal exposure to the cosmetic ingredient, phenoxyethanol, for use in risk assessment. Reg Toxicol Pharmacol.

[CR9] Hiller J, Klotz K, Meyer S, Uter W, Hof K, Greiner A, Göen T, Drexler H (2019). Toxicokinetics of urinary 2-ethylhexyl salicylate and its metabolite 2-ethyl-hydroxyhexyl salicylate in humans after simulating real-life dermal sunscreen exposure. Arch Toxicol.

[CR10] Hiller J, Klotz K, Meyer S, Uter W, Hof K, Greiner A, Göen T, Drexler H (2019). Systemic availability of lipophilic organic UV filters through dermal sunscreen exposure. Environ Int.

[CR11] Jäger T, Eckert E, Leibold E, Bader M (2022). Reliable determination of the main metabolites of 2-phenoxyethanol in human blood and urine using LC–MS/MS analysis. Anal Methods.

[CR12] Jäger T, Eckert E, Leibold E, Bader M (2024). A specific and sensitive GC–MS/MS method for the quantitative determination of 2-phenoxyethanol and selected metabolites in human blood and urine. J Anal Tox.

[CR13] Kim TH, Kim MG, Kim MG, Shin BS, Kim K-B, Lee JB, Paik SH, Yoo SD (2015). Simultaneous determination of phenoxyethanol and its major metabolite, phenoxyacetic acid, in rat biological matrices by LC–MS/MS with polarity switching: application to ADME studies. Talanta.

[CR14] Kwon M, Park JB, Kwon M, Song J, Yeo CS, Bae SH (2021). Pharmacokinetics of 2-phenoxyethanol and its major metabolite, phenoxyacetic acid, after dermal and inhaled routes of exposure: application to development PBPK model in rats. Arch Toxicol.

[CR15] Labat L, Humbert L, Dehon B, Multigner L, Garlantezec R, Nisse C, Lhermitte M (2008). Determination of urinary metabolites of glycol ethers by gas chromatography mass spectrometry. Ann Toxicol Anal.

[CR16] Lidén C, Andersson N, White IR (2022). Preservatives in non-cosmetic products: increasing human exposure requires action for protection of health. Contact Dermatitis.

[CR17] Lockley DJ, Howes D, Williams FM (2005). Cutaneous metabolism of glycol ethers. Arch Toxicol.

[CR18] Multigner L, Ben BE, Arnaud I, Haguenoer JM, Jouannet P, Auger J, Eustache F (2007). Glycol ethers and semen quality: a cross-sectional study among male workers in the Paris Municipality. Occup Environ Med.

[CR19] SCCS (Scientific Committee on Consumer Safety) (2016) Opinion on Phenoxyethanol. SCCS/1575/16. https://health.ec.europa.eu/system/files/2021-08/sccs_o_195_0.pdf. Accessed 19 Jun 2023

[CR20] Troutman JA, Rick DL, Stuard SB, Fisher J, Bartels MJ (2015). Development of a physiologically-based pharmacokinetic model of 2-phenoxyethanol and its metabolite phenoxyacetic acid in rats and humans to address toxicokinetic uncertainty in risk assessment. Reg Toxicol Pharmacol.

[CR21] Viau C, Vyskocil A (1995). Patterns of 1-hydroxypyrene excretion in volunteers exposed to pyrene by the dermal route. Sci Total Environ.

[CR22] Zhang F, LeBaron MJ, Marty MS (2022). Prediction of tissue and urine concentrations of 2-phenoxyethanol and its metabolite 2-phenoxyacetic acid in rat and human after oral and dermal exposures via GastroPlusTM physiologically based pharmacokinetic modelling. SAR QSAR Environ Res.

